# Specific modification at the C-terminal lysine residue of the green fluorescent protein variant, GFPuv, expressed in *Escherichia coli*

**DOI:** 10.1038/s41598-019-41309-8

**Published:** 2019-03-18

**Authors:** Takahiro Nakatani, Norihisa Yasui, Issei Tamura, Atsuko Yamashita

**Affiliations:** Graduate School of Medicine, Dentistry and Pharmaceutical Sciences, Okayama University, 1-1-1, Tsushima-naka, Kita-ku, Okayama, 700-8530 Japan

## Abstract

Green fluorescent protein (GFP) is amenable to recombinant expression in various kinds of cells and is widely used in life science research. We found that the recombinant expression of GFPuv, a commonly-used mutant of GFP, in *E. coli* produced two distinct molecular species as judged by in-gel fluorescence SDS-PAGE. These molecular species, namely form I and II, could be separately purified by anion-exchange chromatography without any remarkable differences in the fluorescence spectra. Mass spectrometric analyses revealed that the molecular mass of form I is almost the same as the calculated value, while that of form II is approximately 1 Da larger than that of form I. Further mass spectrometric top-down sequencing pinpointed the modification in GFPuv form II, where the ε-amino group of the C-terminal Lys238 residue is converted into the hydroxyl group. No equivalent modification was observed in the native GFP in jellyfish *Aequorea victoria*, suggesting that this modification is not physiologically relevant. Crystal structure analysis of the two species verified the structural identity of the backbone and the vicinity of the chromophore. The modification found in this study may also be generated in other GFP variants as well as in other recombinant expression systems.

## Introduction

Green fluorescent protein (GFP), originally discovered in the jellyfish, *Aequorea victoria*^1^, is widely utilized for the fluorescent labeling of any proteins in various cells, since its chromophore is formed by autocatalytic cyclization after the expression and folding^2–4^, without any other molecules in both prokaryotic and eukaryotic cells^5^, and the fluorescent characteristics of GFP remain intact in the form of fusion with other proteins^6^. In addition, many kinds of GFP variants have been developed in which the fluorescence intensity, wavelength characteristics, optimum temperature, and the chromophore formation rate are optimized for various purposes^7^. The application of GFP in the modern life sciences is getting wider; for example, as tools for investigating the intracellular localization of proteins, protein-protein interactions, etc.

In the fields of biochemistry and structural biology, the GFP-fusion system with the protein of interest is often used to evaluate the conditions that are suitable for sample preparation. For example, hydrodynamic states, such as monodispersity, oligomerization, folding and thermostability in solution, could be evaluated by fluorescence-detection size-exclusion chromatography as well as some kind of the native-polyacrylamide gel electrophoresis (PAGE) analyses^8–12^. The SDS-PAGE analysis in which the samples are subjected to without heat denaturation is also useful to assess the molecular weight and integrity of the fusion protein by detection of the fluorescent bands^13–15^. GFP is also utilized for topology mapping of membrane proteins^16^ or protein solubility assessment using split GFP^17^. These methods, based on the fluorescence of GFP, are useful because they allow us to screen the expression constructs, as well as the conditions of the sample preparation for biochemical and structural analyses with high-throughput using a small amount of samples. On the other hand, the biochemical property of the recombinantly expressed reporter GFP itself has not been paid attention to extensively, assuming its explicit protein stability.

Among the wide variety of variants available, GFPuv is the well-known folding mutant of GFP that contains the so-called cycle3 mutations (F99S, M153T, and V163A) with an optimized codon usage for the expression in *Escherichia coli*^18^. In the course of our biochemical study utilizing GFP, we accidentally found that two distinct protein bands of GFPuv were separated on an SDS-PAGE gel after the recombinant expression in *Escherichia coli* under a conventional condition often used in biochemical studies. In the present study, we successfully purified these two molecular species separately and characterized them by various biochemical and structural analyses. We found that one of the molecular species showed the conversion of the ε-amino group into a hydroxyl group in Lys 238 residue at the C terminus, which is conserved in many of the GFP variants used in life science research. The results obtained in this study provided noteworthy information on the utilization of GFP, especially for clarifying the biochemical properties of GFP and its fusion proteins.

## Results

### Two molecular species of GFPuv were generated by recombinant expression in *Escherichia coli*

**Figure 1:**
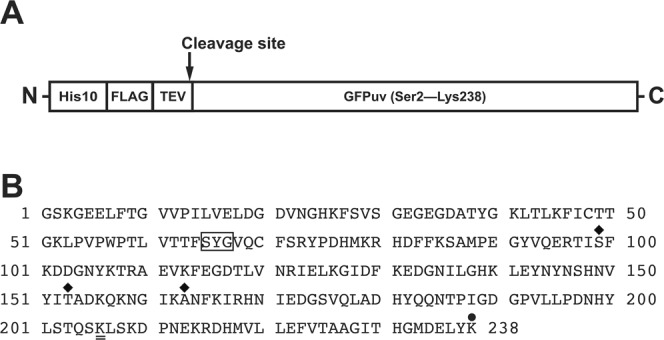
Construction of GFPuv. (**A**) Schematic representation of the expression construct used in this study. (**B**) Amino acid sequence of GFPuv used in this study. The residues forming a chromophore are boxed, the cycle3 mutations (F99S, M153T, and V163A) are labeled with diamonds and the substitution to prevent the dimer formation (A206K) is marked with a double line. The C-terminal Lys residue is labeled with a black circle.

For recombinant expression and purification of green fluorescent protein (GFP), one of the variants of GFP, GFPuv, was used in this study. We designed a GFPuv expression construct in which a sequence consisting of a decahistidine (His10), FLAG tag and TEV protease cleavage site was fused to the N-terminus (HFT-GFPuv) (Fig. 1A). After cleavage of the tag with TEV protease, a GFPuv protein consisting of 238 residues with a substitution of the N-terminal Met residue with Gly residue is generated (Fig. 1B). The GFPuv protein used in this study has the mutation to prevent the dimer formation (A206K) in addition to the cycle3 mutations (F99S, M153T, and V163A)^11,19,20^ (Fig. 1B).

**Figure 2:**
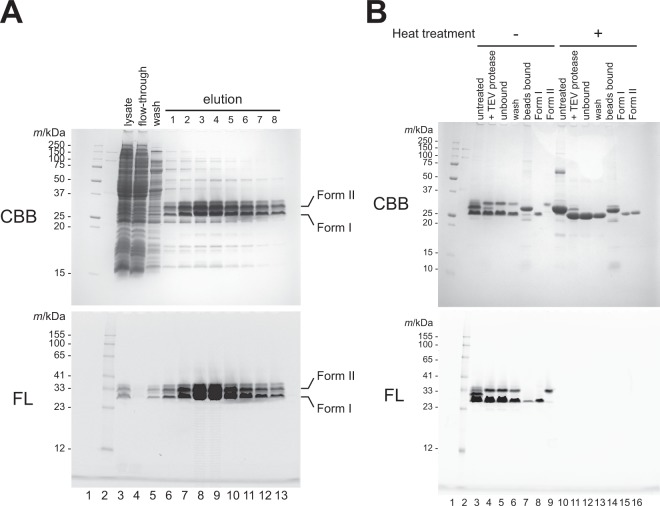
Characterization of recombinant GFPuv protein. (**A**) SDS-PAGE analysis of the fractions from a Ni-NTA column during the purification of HFT-GFPuv. (**B**) Heat treatment of GFPuv form I and form II. Fluorescent bands (shown as “FL”) were visualized under ultraviolet light without any staining processes. After the detection of the fluorescent signal, the same gel was stained with CBB (shown as “CBB”).

HFT-GFPuv construct was expressed in *Escherichia coli* BL21 (DE3) pLysS strain and purified from the soluble fraction using a Ni-NTA column. When the eluted fractions were separated by SDS-PAGE without heating, mainly two bands were detected (Fig. 2A). Both bands emit fluorescence (Fig. 2A, bottom). The species with a higher mobility was named GFPuv form I, and that with a lower mobility was named GFPuv form II. The SDS-PAGE band mobilities of the two GFPuv species were further analyzed after the subsequent purification by the removal of the purification tag (Fig. 2B). In the case of the unheated samples, the mobility differs greatly between GFPuv form I and form II on the gel (Fig. 2B, lanes 8 and 9). On the other hand, almost the same band mobilities were observed after the heat treatment (Fig. 2B, lanes 15 and 16), which suggested that the difference between the mobility of forms I and II under the nondenatured condition was not caused by a cleavage of the polypeptide chain. It should be noted that the separation of GFPuv forms I and II can be observed in some particular polyacrylamide gels with a high resolution for separation, and we did not observe the separation of both forms of GFPuv when using a 15% in-house gel (Supplementary Fig. S1).

### GFPuv forms I and II showed a difference in their isoelectric points

**Figure 3:**
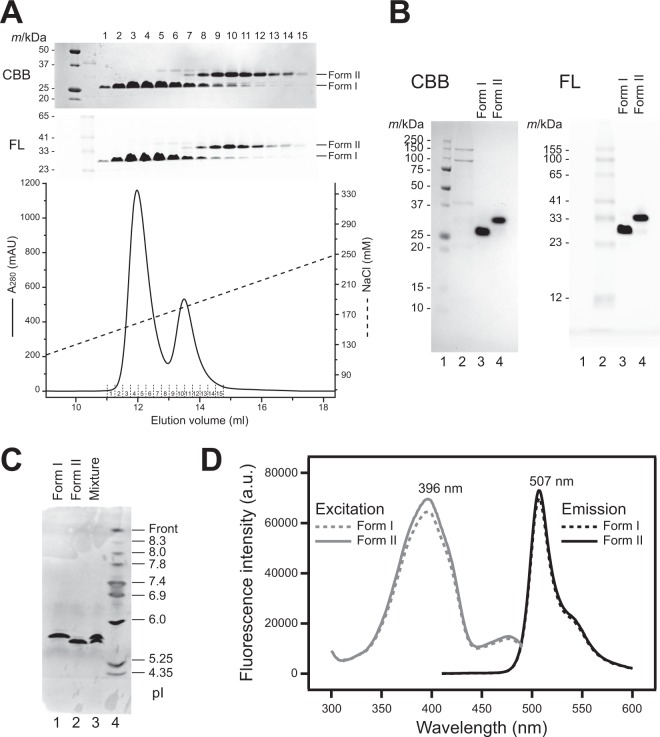
Isoelectric points of GFPuv form I and form II. (**A**) Purification of HFT-GFPuv using a Mono-Q column. The CBB-stained (*top*) and fluorescence-detection (*middle*) SDS-PAGE results for each fractions and the elution profile (*bottom*) are shown. The original uncropped gel images are shown in Supplementary Figure S6. (**B**) SDS-PAGE analysis of GFPuv form I and form II, detected by CBB-staining and fluorescence (FL). (**C**) Isoelectric focusing electrophoresis analysis of the purified GFPuv form I and form I. GFPuv form I (lane 1), form II (lane 2), and their mixture (lane 3) were analyzed with standard proteins (lane 4). Proteins were visualized by CBB staining of the gel. (**D**) Excitation and emission spectra (gray and black lines, respectively) of GFPuv form I (dashed line) and form II (solid line) are shown.

The histidine-tag purified recombinant GFPuv was subjected to anion exchange chromatography. Two major peaks were observed in the elution profile by a linear gradient of NaCl concentration. Each peak corresponded to form I and form II, as judged by SDS-PAGE analysis (Fig. 3A). GFPuv form I was eluted at a lower salt concentration (~158 mM) than form II (~180 mM) (Fig. 3A), which suggested that GFPuv form I and form II have a difference in their surface charges. GFPuv form I and form II were purified almost as a single component by carrying out re-chromatography (Fig. 3B), and the purified samples were used for the subsequent experiments.

In order to estimate their isoelectric points (pI), the purified GFPuv was subjected to isoelectric focusing (Fig. 3C). The pI values of form I and form II were estimated as 5.76 and 5.64, respectively (Fig. 3C and Supplementary Fig. S2). The result is consistent with the elution profile of the anion exchange chromatography at pH 8.0 described above, in which form II is more tightly bound to the resin than form I.

The fluorescence properties of the purified GFPuv form I and form II were investigated. The excitation and emission spectra of both forms were similar, with the maximum peaks at ~396 nm and 507 nm, respectively (Fig. 3D). The observed maximum excitation and emission wavelengths are almost the same as the values reported elsewhere^21,22^, indicating that GFPuv form I and form II share the chromophore with a phenol form^23^ and the almost same fluorescence characteristics with the properly-formed chromophore environment. Relative fluorescence intensities at the excitation and emission peaks were also comparable in both forms (Fig. 3D), suggesting that the molecular brightness of both forms is quite similar. The results suggest that the differences between the two forms give a negligible effect to their chromophore environments, and may not exist in the close vicinity of the chromophores.

### Mass spectrometric analysis revealed that GFPuv form I and form II differ in molecular mass by approximately 1 Da

In order to analyze the differences between GFPuv form I and form II further, molecular masses of intact GFPuv form I and form II were measured using a conventional ESI-MS (Supplementary Fig. S3). The values of the form I and form II were estimated to be 26759.36 and 26760.55, respectively, that is, GFPuv form II showed the molecular mass 1.19 Da larger than that of form I.

**Figure 4:**
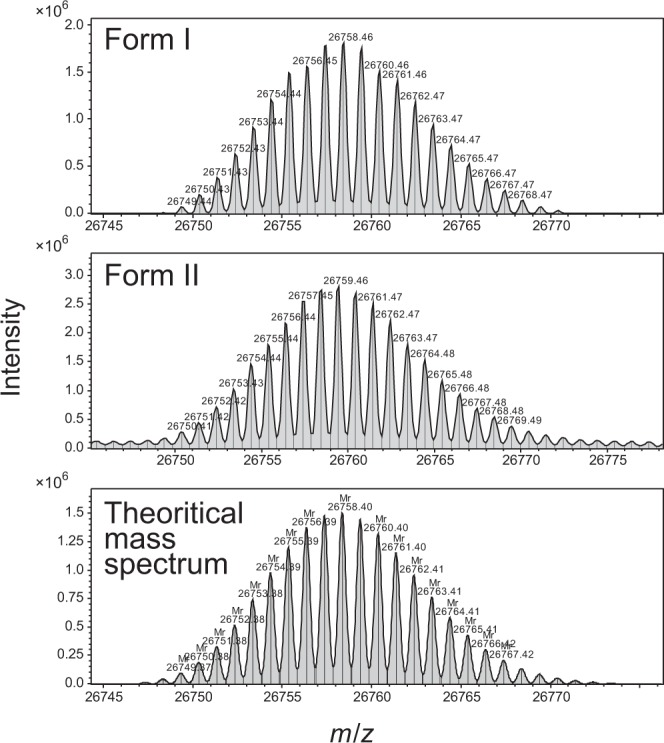
Mass spectra of intact form of GFPuv form I and form II. Mass spectra obtained by ESI-MS measurement for GFPuv form I (*top*) and form II (*middle*) were shown. The theoretical mass spectrum of GFPuv after the formation of the enol form of chromophore is shown (*bottom*).

Mass spectra of GFPuv form I and form II were also obtained by a high-resolution ESI-MS measurement and compared with the theoretical mass spectrum of GFPuv after the formation of the enol form of chromophore (Fig. 4). The differences in the observed mass of form I and the theoretical one for GFPuv were quite small, and its isotope pattern was also similar to that of the theoretical mass spectrum. On the other hand, the isotopic patterns for form II did not match, and the estimated mass showed about 1 Da over the theoretical molecular mass (26742.3533). It should be noted that the observed mass difference was unlikely caused by the difference in protonation state of the hydroxyl group of Tyr66 forming the chromophore^23^, because the samples were detected as the proton adduct ions regardless of the protonation state of each amino acid, including Tyr66, due to the usage of acidic mobile phases for separation by HPLC.

The mass spectrometric data described above indicate that the difference between the mobility in SDS-PAGE and the isoelectric point of GFPuv form I and form II are not caused by cleavage of the polypeptide chain.

### Approximately 1 Da molecular mass increase in GFPuv form II was attributed to the conversion of the ε-amino group of the C-terminal Lys238 residue into the hydroxyl group

**Figure 5:**
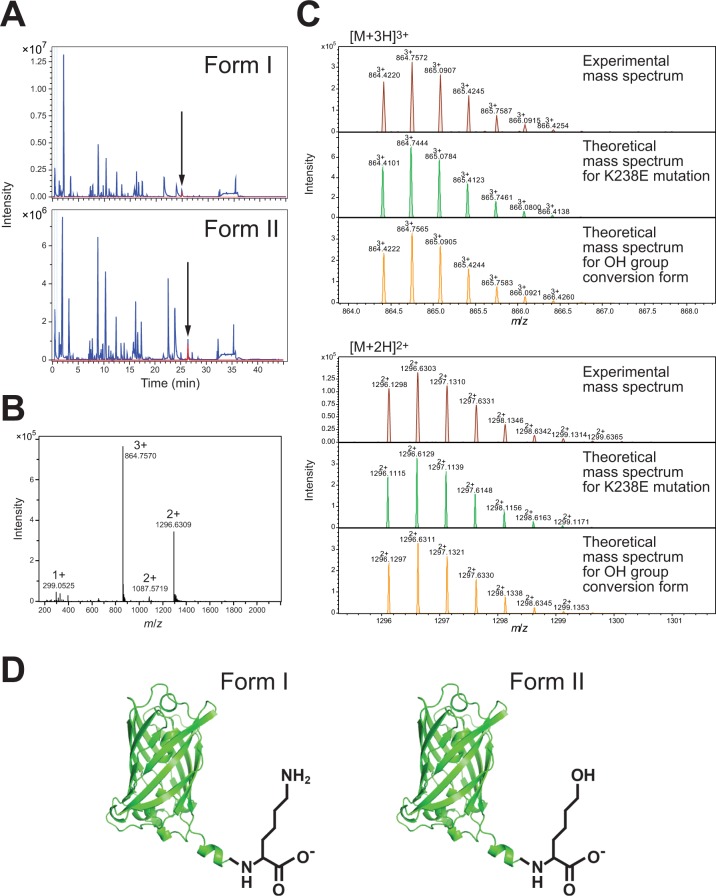
Determination of the modification type and site in GFPuv form II. (**A**) Liquid chromatography profiles of trypsin digests of GFPuv form I (*top*) and form II (*bottom*). A pair of peaks showing the retention time difference in GFPuv form I and form II digests is indicated by arrows. (**B**) ESI-MS spectrum of the H_231_GMDELYK_238_ fragment ions from GFPuv form II. (**C**) Comparison of the observed mass of the H_231_GMDELYK_238_ fragment ions from GFPuv form II with the theoretical mass spectra for the fragment containing the candidate modifications. (**D**) Schematic illustration of GFPuv form I and form II.

Since the observed mass differed by about 1 Da from the theoretical value, it was suspected that some types of posttranslational modification, accompanied with a mass difference, occurred in GFPuv form II. To identify the modified residues, we further analyzed GFPuv form I and form II by top-down sequencing (TDS) using MALDI In-Source-Decay (MALDI-ISD). The analysis of trypsin digests of GFPuv form I and form II with liquid chromatography revealed that the retention time of almost all of the peaks for digests from form II was shared with those from form I, whereas a pair of peaks showing the retention time difference between form I and form II was observed (Fig. 5A, arrow). Mass spectra of all peaks on LC revealed that the peaks with different retention times in GFPuv form I and form II correspond to the H_231_GMDELYK_238_ fragment, whereas the observed molecular mass of the other fragments was shared in both forms of GFPuv. TDS of the H_231_GMDELYK_238_ fragment from GFPuv form II revealed that the mass of Lys238 residue increased by approximately 1 Da (Supplementary Fig. S4).

By searching for the database of protein modifications for mass spectrometry (UNIMOD)^24^, the substitution of Lys238 with Glu residue and the conversion of the ε-amino group of Lys238 into a hydroxyl group (conversion of lysine to 6-hydroxy-l-norleucine) were proposed as candidates accounting for a modification accompanied by mass increase of 1 Da in the sequence of the H_231_GMDELYK_238_ fragment. Therefore, we compared the observed mass of the H_231_GMDELYK_238_ fragment ions from GFPuv form II (Fig. 5B) and the theoretical mass spectra for the fragments of the two candidates, the Lys residue substitution with Glu residue or the conversion to 6-hydroxynorleucine (shown as “OH group conversion form”) (Fig. 5C). The difference from the theoretical molecular mass of the fragment containing the K238-6-hydroxynorleucine conversion (−0.0002/0.0001 Da) was smaller than the difference from the theoretical mass of the K238E substitution (0.0119/0.0183 Da). Furthermore, the mass spectrum of the H_231_GMDELYK_238_ fragment ions from GFPuv form II was also better matched to the theoretical mass spectrum of the fragment containing the Lys238 converted to 6-hydroxynorleucine, in terms of the isotope pattern of ions (Fig. 5C). The results of the mass spectrometric analysis, described above, indicated that the mass increase of ~1 Da in GFPuv form II is most likely caused by the conversion of an amino group into a hydroxyl group occurred in the side chain of Lys238 residue at the C terminus (Fig. 5D). The modification is in accord with the observed pI difference between the forms I and II.

### The modified form of GFP is not dominant in the body of the jellyfish

**Figure 6:**
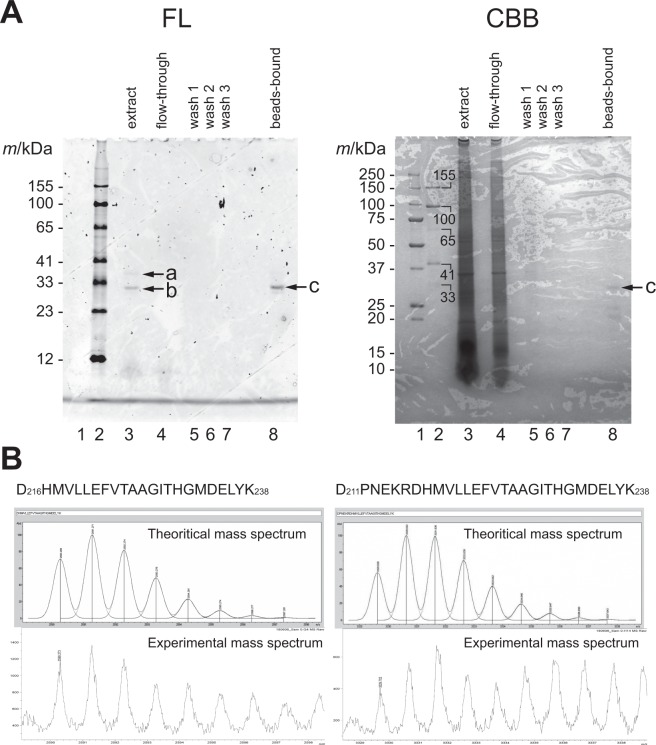
Extraction and mass spectrometric analysis of the native GFP from jellyfish. (**A**) SDS-PAGE analysis of the crude extract and the fractions of the purification processes. Fluorescent bands were visualized under ultraviolet light without any staining processes (*left*). After the detection of fluorescent signals, the same gel was stained with CBB (*right*). (**B**) MS spectra of the trypsinized fragment ions from GFP. Experimental mass spectrum (*bottom*) and theoretical mass spectrum (*top*) are shown for two fragment ions.

Next, we sought to investigate whether the modification in the C-terminal Lys residue of the recombinant GFPuv is also found in the native GFP in the photogenic organs of a jellyfish so as to verify the physiological significance of this modification. To this end, we extracted GFP from the jellyfish *Aequorea victoria* and analyzed it on SDS-PAGE (Fig. 6A). In the crude extraction (Fig. 6A, right, lane 3), the fluorescent band with the mobility reasonable with the calculated molecular weight of GFP (indicated with b), as well as the additional faint fluorescent band (indicated with a), were detected under UV illumination. In the concentrated and purified GFP by the immuno-precipitation with anti-GFP antibody, only the major species (indicated with c) was detected under UV illumination, while the species corresponding to the faint fluorescent band (band a) was not effectively concentrated. It was confirmed that the dominant molecular species were highly purified and separated from other impurities on the gel by visualization with CBB staining (Fig. 6A, right).

In order to analyze the modification, in-gel digestion of the protein band c with trypsin followed by mass spectrometric analysis was carried out. The isotope patterns of two identified ionized fragments containing C-terminal Lys238 residue were matched to the theoretical ones of the fragments containing unmodified Lys238 residue (Fig. 6B). Therefore, we concluded that the majority of native GFP exist as an unmodified form in the jellyfish photogenic organ in terms of the C-terminal residue. Therefore, the modification in form II found in this study likely generated under the condition of the recombinant expression in *E. coli*.

### Crystal structures of GFPuv form I and form II

**Figure 7:**
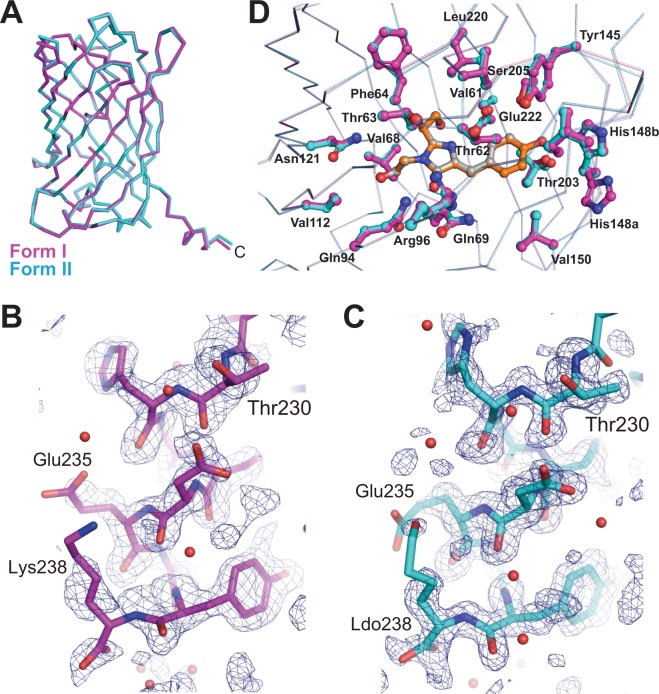
Crystal structures of GFPuv form I and form II. (**A**) Overall structure of GFPuv form I (magenta) and form II (cyan) are superimposed. (**B**,**C**) Close-up view of the C-terminal region of GFPuv form I (**B**), form II (**C**). The C-terminal α-helices are shown in stick models, and the simulated annealing-omit electron density maps at 1.9 σ are shown in dark blue. (**D**) Close-up view of the region surrounding the chromophore. The chromophores and the side chains of the amino acid residues around the chromophore within 4 Å are shown in the ball and stick model. Colors for GFPuv form I and form II are same to those in A. The chromophore of GFPuv form I and form II are colored in orange and gray, respectively. All structure images were prepared using PyMOL (http://www.pymol.org).

We determined the crystal structures of GFPuv form I and form II at 1.65 Å and 1.28 Å resolution, respectively (Table 1, Fig. 7A). Both models contain almost all residues of GFPuv including 9 residues in the C-terminal flexible region spanning Thr230–Lys238 (Fig. 7B,C), though we omitted N-terminal three residues (Gly1–Lys3) from the final model due to the low quality of the electron-density map. The final model of GFPuv form II includes 6-hydroxy-l-norleucine (LDO) at the C-terminus instead of lysine residue.

**Table 1:** Crystallographic Data and Refinement Statistics.

	GFPuv form I	GFPuv form II
**Data collection**		
Space group	*P*2_1_	*P*2_1_
Unit cell dimension		
*a*, *b*, *c* (Å)	47.6, 51.0, 47.3	47.3, 51.2, 48.5
β (°)	98.5	100.8
Resolution (Å)	50.0–1.64 (1.68–1.64)	50.0–1.28 (1.30–1.28)
Total reflections	653331	367612
Unique reflections	26115 (1282)	56911 (2566)
Completeness (%)	95.7 (96.5)	96.3 (87.4)
*R*_sym_ (%)	5.9 (51.7)	7.1 (24.8)
<*I*/σ(*I*)>	27.4 (1.8)	27.8 (7.2)
**Refinement**		
*R*_work_	0.188 (0.291)	0.175 (0.195)
*R*_free_	0.216 (0.322)	0.195 (0.201)
Number of atoms	2051	2292
Protein	1846	1886
Ligand/ion	31	38
Water	174	368
B-factors	32.9	16.8
Protein	32.1	14.5
Ligand/ion	31.9	15.9
Water	41.8	28.6
R.m.s. deviation		
Bond lengths (Å)	0.007	0.006
Bond angles (°)	1.08	1.14
Ramachandran plot		
Favored (%)	99.1	98.7
Allowed (%)	0.9	1.3
Outliers (%)	0	0

The overall structures of GFPuv form I and form II are almost identical when these are superposed, showing 0.145 Å rmsd for Cα atoms of 233 residues (Fig. 7A), though slight differences arise mainly in the conformations of the turns consisting of Asn23–Gly24 and Asp155–Lys158 (Fig. 7A). The C-terminal α-helices of GFPuv form I and form II also show almost the same structure in each other, except for the slight differences in the side chains of the C-terminal residue and the adjacent Glu235, where the electron densities at the end of the substituent groups in form II are more disordered than those in form I (Fig. 7B,C). As far as we inspected the data at the current resolutions, no apparent modification was observed in the other regions of the crystallographic structures.

The regions around the chromophore of both forms show almost the same structure, although the alternative conformers of the side chains of His148 and Thr203 could be modeled in GFPuv form I and form II, respectively (Fig. 7D). These observations support the fact that there is no large difference in the fluorescence characteristics between GFPuv form I and form II.

## Discussion

Chromophore formation of green fluorescent protein (GFP) is a built-in type of posttranslational modification, i.e., the chromophore of GFP forms by an autocatalytic reaction of the backbone involving cyclization, oxidation, and dehydration reactions^7^. Here, we demonstrated that the second posttranslational modification is generated at the side chain of the C-terminal Lys238 residue of the GFPuv, a mutant of GFP, when over-expressed in *E. coli* BL21 (DE3) pLysS strain.

The mass spectrometric data supports that the conversion of an amino group into a hydroxyl group occurs in the ε-amino group of the Lys238 residue (Fig. 5). This modification causes a decrease in the net charge of the modified form of GFPuv (named form II) from that of the unmodified GFPuv (form I) at the neutral pH. In addition, the charge difference occurs on the molecular surface, not the interior of the β-barrel structure, because the Lys238 residue locates on the solvent accessible α-helix. Several lines of biochemical evidence also support this difference in the net surface charge of GFPuv molecules. In particular, GFPuv form II is eluted at a higher NaCl concentration than GFPuv form I in the anion-exchange chromatography at pH 8.0 (Fig. 3A), and the pI value of GFPuv form II is lower (0.12) than that of GFPuv form II (Fig. 3C). The conversion of the side chain of Lys238 also affects the mobility on SDS-PAGE gel when the samples are analyzed without heat denaturing treatment (Figs 2A,B and 3B). The binding of SDS to GFPuv molecules may be affected by this side chain modification.

It is thought that the modified form of GFP is rarely generated in the body of the jellyfish *Aequorea victoria*, when the major species from the jellyfish extract were analyzed with mass spectrometry. Form II of GFPuv is generated in *E. coli* cells, or during the cell lysis, because the two main fluorescent bands, corresponding to GFPuv forms I and II, are detected in the lysate (Fig. 2A, lane 3). It is highly possible that the generation of the form II occurs under specific conditions during the recombinant expression in *E. coli* cells, such as the redox conditions that are favorable for the reactions, and the existence of intrinsic enzymes catalyzing the reactions, which will be discussed below. It is still unclear whether this modification occurs in other host cells, such as yeast and mammalian cells, rather than *E. coli* cells, when GFPuv is over-expressed. GFP and most of its mutants, including GFPuv, share the amino acid sequence at the C-terminal α-helix region including the Lys238 residue (Supplementary Fig. S5). Therefore, the similar conversion of a side chain that is found in the GFPuv molecule could be generated in other variants of GFP, when overexpressed in *E. coli* or other host cells; however, this possibility needs to be verified in the future.

There are 21 Lys residues in the GFPuv molecules used in this study (Fig. 1B). The modification found in this study is quite specific to the side chain of Lys238 residue. Although it has not been investigated yet, it may be important for this specific modification to occur that Lys residue exists exactly at the C-terminus of a flexible α-helix. We first suspected that some kinds of enzymes are involved in the generation of this modification in *E. coli* because the Lys238 of GFPuv is modified with the extremely high specificity, and it is unlikely that this modification occurs spontaneously. However, there are no reported enzymes that are directly involved in the conversion of the amino group of the Lys side chain into the hydroxyl group in *E. coli*, as far as we know. On the other hand, it has been reported that the formation of aminoadipic semialdehyde, an oxidized product of lysine and a precursor of its reduction product 6-hydroxynorleucine, was observed in proteins from a tissue sample as well as culture cells^25^. The proposed reaction mechanism of this conversion is the multi-step scheme, in which the metal-catalyzed oxidation of the side chain of Lys generates aminoadipic semialdehyde, while further reaction is needed to reduce the 6-oxo group to the hydroxyl group to generate 6-hydroxynorleucine^25^. A similar mechanism may be true, at least in part, for the case of the modification of Lys238 of GFPuv. In the case of GFPuv, hydrogen peroxide molecules generated during the process of auto-catalyzed formation of chromophore^26^ may be involved in the oxidation/reduction reactions. Elucidation of the mechanism of the specific modification of C-terminal Lys residue in GFPuv will be the subject in the future study.

The fluorescent properties (Fig. 3D), the overall structure (Fig. 7A) as well as the structure in the vicinity of chromophore (Fig. 7D) are almost the same in GFPuv form I and form II, although their chemical composition at the C-termini is different. These seem to be due to the fact that the modification site is ~30 Å away from the chromophore. The difference between form I and form II in pI value is also not significant (0.12) (Fig. 3C). Therefore, even if both forms are present in the system, there is almost no influence on the results obtained by using GFP and its variants in the cases where GFP is utilized just as a fluorescent label. However, it seems necessary to take it into consideration for the interpretation of the results for investigations of folding and physicochemical properties of the proteins of interest using GFP as a reporter protein, although differences in chemical and physical properties between both forms are small. For example, in the case of evaluating the quality of GFP fusion proteins for structural studies by SDS- and native-PAGE analyses, doublet bands may appear regardless of the quality of the target proteins, which affects the accuracy in our data interpretation. The next issue to be clarified is under which expression conditions the same modification is generated, for example, whether it is also introduced in GFP fusion proteins.

## Methods

### DNA Manipulations

A DNA fragment encoding GFPuv, which has a decahistidine (His10), FLAG tag, TEV protease cleavage site, was generated by a three-step extension PCR. In the first PCR, pCGFP-BC^11^ was used as a template and the following primer set was utilized: 5′-AAGGTGAAAACCTGTACTTCCAGGGATCCAAAGGAGAAGAACTTTTCAC-3′ (forward) and 5′-GCTGGCTAGCTCATTTGTAGAGTTCATCCATGC-3′ (reverse). In the second and third PCR, 5′-GCAGCGACTACAAAGACGACGATGACAAAGGTGAAAACCTGTACTTCC-3′, and 5′-ATACCCATGGCCCACCACCACCACCATCATCATCATCATCACAGCAGCGACTACAAAGACGAC-3′, respectively, were used as forward primers. The reverse primer was shared with the first PCR. The resulting DNA fragment was inserted into pCGFP-BC, a pET25b (Novagen) based vector, using the NcoI and NheI sites to make pHFT-GFPuv. This expression vector was verified by DNA sequencing on a 3500 Genetic Analyzer (Applied Biosystems).

### Expression and Purification of GFPuv

The *E. coli* BL21 (DE3) pLysS strain [F- *omp*T *hsd*S_B_(r_B_^−^ m_B_^−^) *gal dcm* (DE3) pLysS (Cam^R^)] (Novagen) was transformed with pHFT-GFPuv and was cultured in LB medium (Nacalai Tesque) containing 34 µg/mL chloramphenicol and 50 µg/ml carbenicillin at 37 °C until the OD_600_ value reached to ~0.6. Protein expression was induced by adding 0.1 mM isopropyl-β-d-thiogalactopyranoside, followed by further culture at 20 °C overnight. Cells were harvested by centrifugation and frozen at −80 °C until needed.

Cells were lysed by sonication in 20 mM Tris-HCl, 500 mM NaCl, pH 8 on ice. The soluble fraction was obtained as the “lysate” by centrifugation at 17,000 *g* for 30 min at 4 °C and applied to a Ni-NTA agarose column (QIAGEN). After washing the beads with 20 mM Tris-HCl, 500 mM NaCl, 50 mM imidazole, pH 8, proteins were eluted with 20 mM Tris-HCl, 500 mM NaCl, 500 mM imidazole, pH 8. The tagged GFPuv protein was treated with His-tagged TEV protease^27^ at room temperature overnight in a dark and dialyzed against 20 mM Tris-HCl, 500 mM NaCl, pH 8 to remove imidazole. The dialyzed sample was applied to a Ni-NTA agarose column, and the unbound fraction was collected. The column was washed with 20 mM Tris-HCl, 500 mM NaCl, pH 8 to collect the residual proteins.

Separation of GFPuv form I and form II was carried out by anion-exchange chromatography on Mono Q 5/50GL column (GE Healthcare Life Sciences). The column was equilibrated with 20 mM Tris-HCl, pH 8.0, 50 mM NaCl, and elution was performed by a linear gradient from 50 mM to 280 mM NaCl over a 20-column volume. The fractions containing GFPuv form II were pooled and further purified by the same procedure to increase the purity.

### Sodium Dodecyl Sulfate-Polyacrylamide Gel Electrophoresis (SDS-PAGE)

The protein solutions were mixed with SDS-PAGE sample buffer (final concentration of components: 2% SDS, 10% (w/v) glycerol, 0.002% bromophenol blue, 50 mM Tris-HCl, pH 6.8) to prepare the samples for SDS-PAGE analysis. To denature the proteins, the mixed solution was heated at 95 °C for 5 min. Samples were subjected to SDS-PAGE using SuperSep Ace precast gels (Wako Pure Chemical Industries, Japan) and the running buffer (25 mM Tris, 192 mM glycine, 0.1% (w/v) SDS). 15% gels were used unless otherwise indicated. Visualization of fluorescent bands and the image capturing were carried out on a ChemiDoc XRS + Imaging System (BIO-RAD) equipped with a bandpass filter for GFP detection (520 nm, full-width half-maximum [FWHM] = 20 nm, Bio-Rad) under UV illumination. For in-gel fluorescent detection, BenchMark™ Fluorescent Protein Standard (ThermoFisher, product # LC5928) was used as marker proteins. After detection of the fluorescent bands, the gels were stained with CBB Stain One (Nacalai Tesque). Gel image was captured using a ChemiDoc XRS + Imaging System.

### Isoelectric Focusing

Isoelectric focusing of GFPuv was carried out on a PhastSystem using phastgel IEF 3–9 (GE Healthcare Life Sciences) according to the manufacturer’s protocol. Isoelectric points of GFPuv form I and form II were estimated using standard proteins (IEF Marker 3–10, Catalog # 39212-01, ThermoFisher).

### Measurements of Fluorescence Spectra

GFPuv form I and form II were dialyzed against 20 mM Tris-HCl, 150 mM NaCl, pH 7.5. The protein concentration of GFPuv form I and form II were estimated by BCA assay (BCA Protein Assay Kit, ThermoFisher) using BSA as a standard protein. The excitation and emission spectra of GFPuv forms I and II, at a concentration of 0.239 µg/mL in dialysis buffer were obtained at 25 °C using a FluoroMax4 spectrofluorometer (Horiba). The excitation spectrum was measured at a fixed emission wavelength of 507 nm, with a 3 nm bandpass slit. The emission spectrum was measured at a fixed excitation wavelength of 395 nm, with a 3 nm bandpass slit. Fluorescence intensities corrected with the hardware-specific correction parameters and the lamp-output intensities were plotted. The average of the spectra of the several individually diluted GFPuv form I and form II samples (*n* = 5–9) were shown in Fig. 3D.

### Mass Spectrometric Analysis of the Recombinant GFPuv

An electrospray ionization-mass spectrometry (ESI-MS) analysis of intact GFPuv forms I and II was carried out, using an HPLC-Chip/QTOF (G6520 and G4240, Agilent Technologies, Santa Clara, CA, USA). The desalted samples were injected after dilution with 50% acetonitrile with 0.1% formic acid (~1 µM). The deconvoluted mass spectra are shown in Fig. S3. The mass spectra of intact form of GFPuv (Fig. 4) were obtained using the Impact II UHR-TOF MS System (Bruker), coupled to an Agilent 1290 UHPLC. Proteins were applied to Aeris WDEPORE C4, 2.1 × 150 mm (Phenomenex). Eluent A was 0.1% trifluoroacetic acid and eluent B was acetonitrile containing 0.1% trifluoroacetic acid. Proteins were eluted with a linear gradient from 5% to 95% B for 13 min, including a regeneration step at 95% B for 2 min, and an equilibration step at 5% B. To digest the intact samples, the reduced-alkylated GFPuv was mixed with trypsin (Promega) and incubated at 37 °C for 24 h. The digested peptide solutions were analyzed using an Impact II UHR-TOF MS System that was coupled to a UltiMate3000 RSLCnano. Peptides were separated on Acclaim PepMap RSLC C18 2 µm, 0.075 × 500 mm/PepMap 100 C18 5 µm, 0.3 × 5 mm (Dionex). Eluent A was 0.1% trifluoroacetic acid and eluent B was acetonitrile containing 0.1% trifluoroacetic acid. Peptides were eluted with 2 segment linear gradients from 0% to 2% B for 5 min and from 5% to 35% B for 30 min including a regeneration step at 95% B for 3 min and an equilibration step at 2% B. Sequence analysis of the peptides from GFPuv forms I and II were carried out by top-down sequencing using MALDI In-Source-Decay (ISD). ISD data were acquired by using 2,5-dihydroxybenzoic acid as a matrix, and they were analyzed by using Bio Tools software (Bruker). Unimod^24^ was referred to for annotating the modification.

### Preparation and Characterization of the Native GFP in Jellyfish *Aequorea victoria*

The ring canals, with the adjacent tissue of jellyfish *Aequorea victoria* (the generous gift from Mr. Motoki Kawasaki at Kyoto Aquarium), where the photogenic organs exist^28^, were peeled off with tweezers or a razor under a UV illuminator. The tissue was ground in a tube with a homogenizer pestle, mixed with almost the same volume of CytoBuster™ Protein Extraction Reagent (Novagen), and incubated at room temperature overnight. After centrifugation (15,000 *g*, 15 min, 4 °C), the supernatant was collected and dialyzed against 20 mM Tris-HCl, 150 mM NaCl, pH 8.0 and the insoluble materials were removed by centrifugation (15,000 *g*, 15 min, 4 °C). The supernatant containing GFP was concentrated with a concentrator and mixed with the CNBr-activated Sepharose 4B (GE Healthcare Life Sciences) coupled with the anti-GFPuv IgG (produced by Mikuri Immunological Lab. Co., Yao, Osaka, Japan) with gentle rotation at 4 °C for 2 h. The beads were washed with 20 mM Tris-HCl, 150 mM NaCl, 0.05% Tween 20, pH 8.0, three times. After washing the beads, the proteins on the beads were eluted by incubation with SDS-PAGE sample buffer at room temperature for 10 min. The proteins were subjected to SDS-PAGE, as described above. The band corresponding GFP (band c in Fig. 6A,B) was extracted from the gel stained with CBB Stain One (Nacalai Tesque).

In-gel digestion of the extracted protein was carried out with 20 ng/µL trypsin (Promega) in 40 mM ammonium bicarbonate by incubation at 37 °C for 24 h after reduction with 10 mM dithiothreitol, 40 mM ammonium bicarbonate and subsequent alkylation with 55 mM iodoacetamide, 40 mM ammonium bicarbonate. The digests were eluted by addition of 50% acetonitrile with 5% formic acid to the gel. The eluate was concentrated by evaporation.

The digested peptides were analyzed on an Ultraflextreme MALDI-TOF/TOF (Bruker) coupled with the EASY-nLC system (Thermo Fisher Scientific). Peptides were separated on L-Column2 ODS, 2 µm. Eluent A was 0.1% trifluoroacetic acid, and eluent B was acetonitrile containing 0.1% trifluoroacetic acid. Peptides were eluted with a linear gradient from 5% to 40% B for 60 min including a regeneration step at 95% B for 3 min and an equilibration step at 5% B. Obtained data was analyzed by Compass Isotope Pattern software (Bruker).

### X-ray Crystallography

The GFPuv form I and form II samples, purified on an anion-exchange column, were concentrated using Vivaspin6 (MWCO 10,000) to 28.0 mg/ml and 30.5 mg/ml, respectively. Crystals of GFPuv form I and form II were obtained by the sitting-drop vapor-diffusion method at 20 °C in 30% (w/v) PEG 1500, 3% (v/v) MPD, 0.2 M magnesium sulfate, 0.1 M sodium acetate/acetic acid pH 5.5, and 0.2 M ammonium sulfate, 0.1 M MES pH 6.5, 30% PEGMME 5000, respectively. Crystals were fished out from the plate and directly flash-frozen in liquid nitrogen. Diffraction data were collected at a wavelength of 1.0 Å on the beamline BL41-XU of SPring-8 (Harima, Japan) using a PILATUS3 6 M detector (DECTRIS). Data sets were processed with HKL2000^29^. The structures of the GFPuv form I and form II were determined by the molecular replacement method using the atomic coordinates of GFP mutant F99S/M153T/V163A (PDB ID: 1B9C)^30^ as a search model by the program MOLREP^31^. The GFPuv models were manually rebuilt using Coot^32^. Crystallographic refinement was carried out using REFMAC5^33^ and phenix.refine^34^. The qualities of the final models were validated using MolProbity^19^. Data collection and refinement statistics are summarized in Table 1. The coordinates and structure factor amplitudes of GFPuv form I and form II have been deposited in the Protein Data Bank as entries 6IR6 and 6IR7, respectively.

## Supplementary information

Supplementary Information
