# Supplementary material for: Specific modification at the C-terminal lysine residue of the green fluorescent protein variant, GFPuv, expressed in Escherichia coli
Source: Sci Rep. 2019 Mar 18;9:4722. doi: 10.1038/s41598-019-41309-8 (PMC6423240; doi:10.1038/s41598-019-41309-8)
Supplement: Supplementary file 1 — Supplementary Information [file 41598_2019_41309_MOESM1_ESM.pdf]

Supplementary Information for:

Specific modification at the C-terminal lysine residue of the green fluorescent protein variant, GFPuv, expressed in *Escherichia coli*

Takahiro Nakatani, Norihisa Yasui, Issei Tamura, Atsuko Yamashita

Graduate School of Medicine, Dentistry and Pharmaceutical Sciences, Okayama University, 1-1-1, Tsushima-naka, Kita-ku, Okayama, 700-8530, Japan

Corresponding Authors

Norihisa Yasui
E-mail: nyasui@okayama-u.ac.jp

Atsuko Yamashita
E-mail: a_yama@cc.okayama-u.ac.jp

CONTENTS

Supplementary Figure S1. Gel dependency of the separation of GFPuv form I and form II by SDS-PAGE.

Supplementary Figure S2. Estimation of the isoelectric point (pI) of GFPuv form I and form II using standard proteins.

Supplementary Figure S3. ESI-MS spectra of GFPuv form I and form II.

Supplementary Figure S4. Top-down sequencing of the $H_{231}GMDELYK_{238}$ fragment of GFPuv form II using MALDI In-Source-Decay.

Supplementary Figure S5. Structure-based amino acid sequence alignment of GFP and its related proteins.

Supplementary Figure S6. The original gel images displayed on Figure 3A.

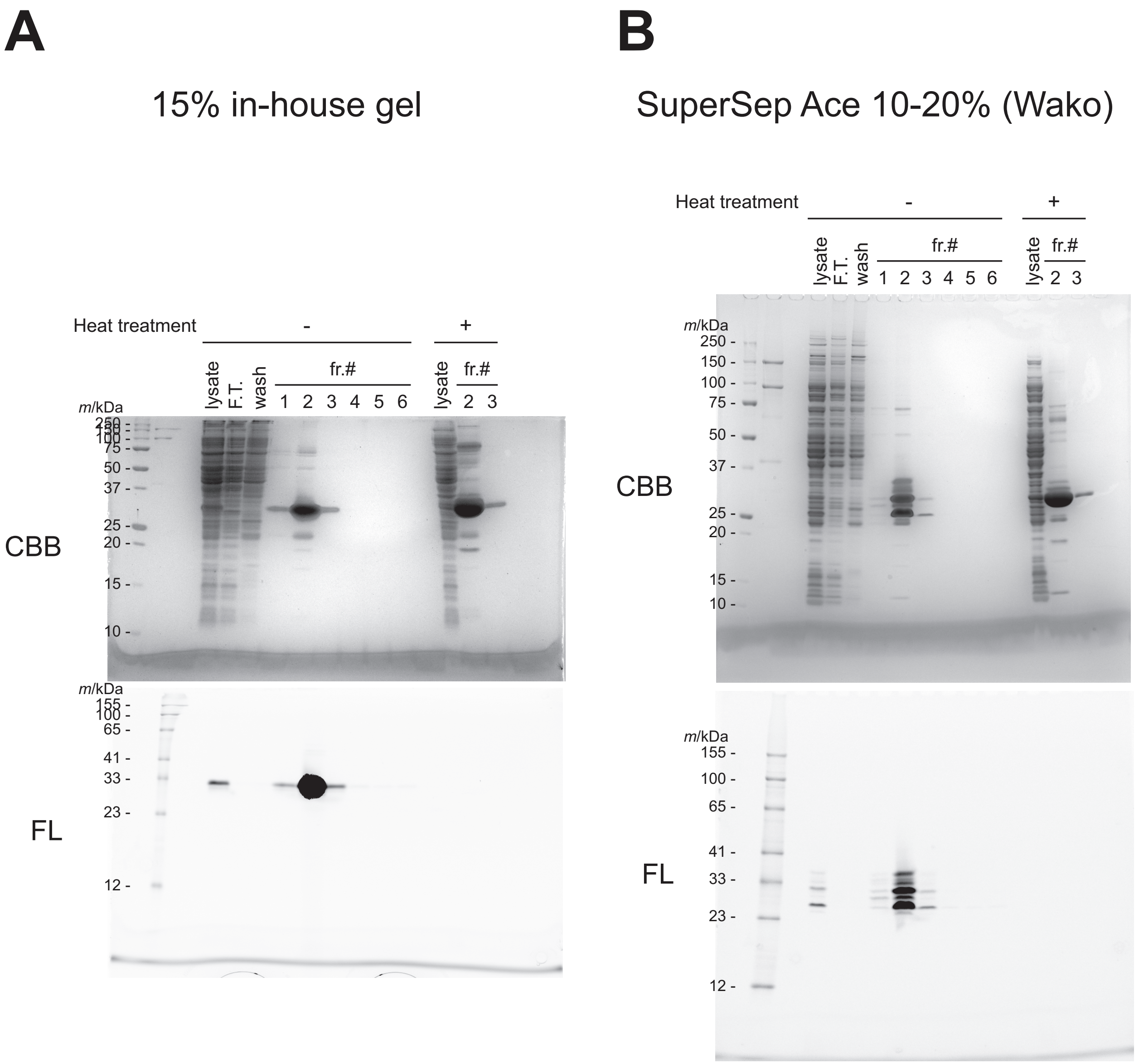

Supplementary Figure S1. Gel dependency of the separation of GFPuv form I and form II by SDS-PAGE. SDS-PAGE analysis of the fractions from a Ni-NTA column during the purification of HFT-GFPuv on a 15% in-house gel (A) and a precast 10–20% gradient gel (B).

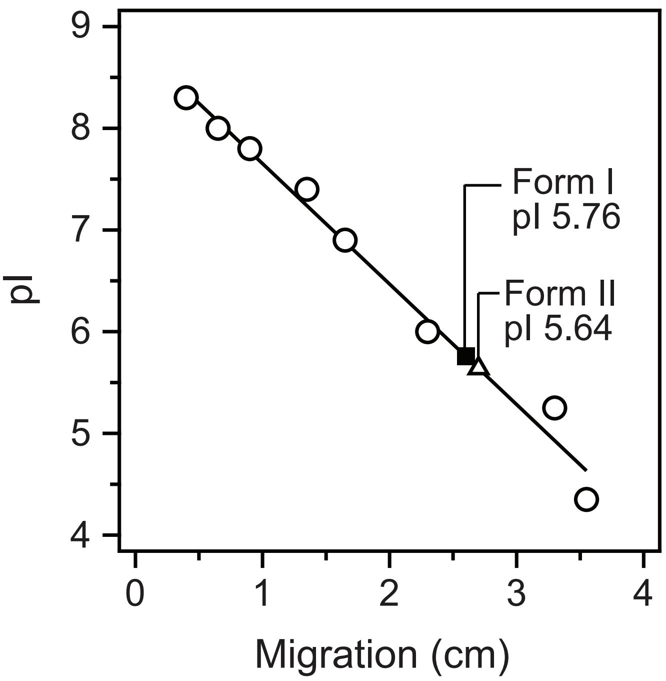

Supplementary Figure S2. Estimation of the isoelectric point (pI) of GFPuv form I and form II using standard proteins. pIs of the standard proteins are plotted against their migration distance from the cathode on the gel in Fig. 3C. Cytochrome c from pig heart (pI 10.7), ribonuclease A from bovine pancreas (pI 9.5), a lectin from *Lens culinaris* (pI 8.3, 8.0, 7.8), myoglobin from horse muscle (pI 7.4, 6.9), carbonic anhydrase from bovine erythrocytes (pI 6.0), β-lactoglobulin from bovine milk (pI 5.3, 5.2), trypsin inhibitor from soybean (pI 4.5), glucose oxidase from *Aspergillus niger* (pI 4.2), amyloglucosidase from *Aspergillus niger* (pI 3.5). The closed square and open triangle indicates the position of GFPuv form I and form II, respectively.

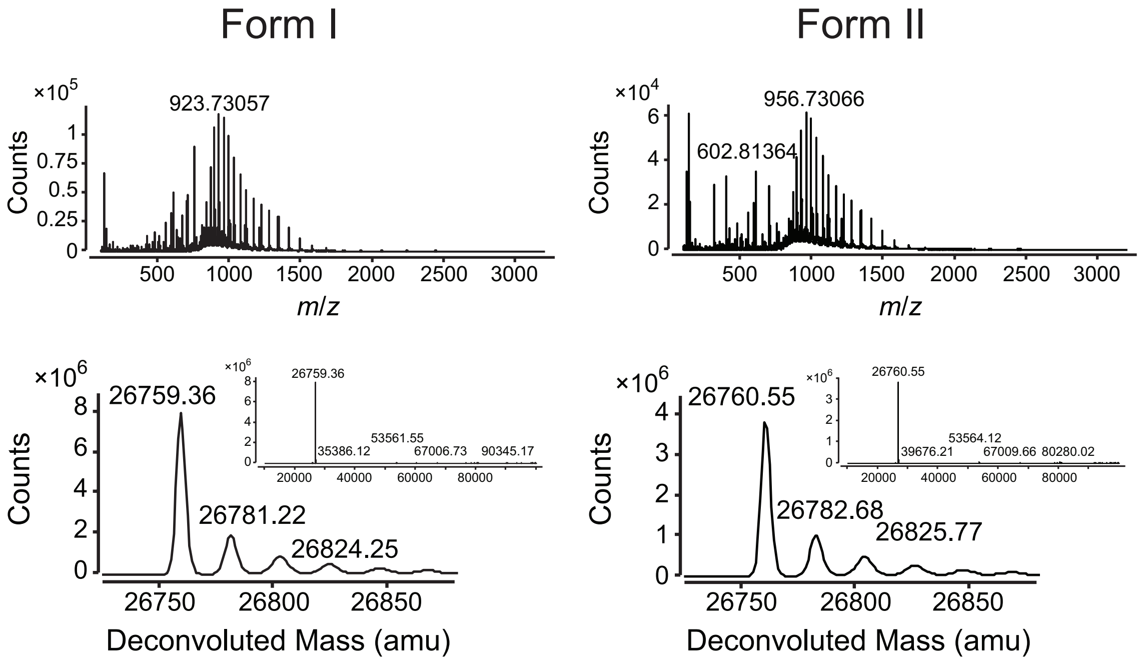

Supplementary Figure S3. ESI-MS spectra of GFPuv form I and form II. Mass spectra (*top*) and deconvoluted mass spectra (*bottom*) of the intact GFPuv form I and form II are shown. Estimated mass values of form I and form II are 26759.36 and 26760.55, respectively.

[M+3H]$^{3+}$

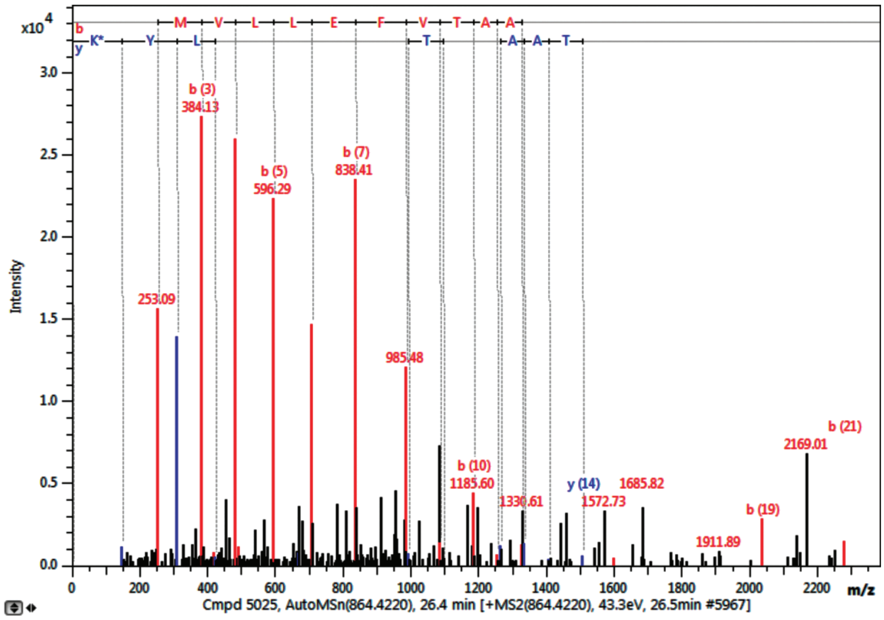

[M+2H]$^{2+}$

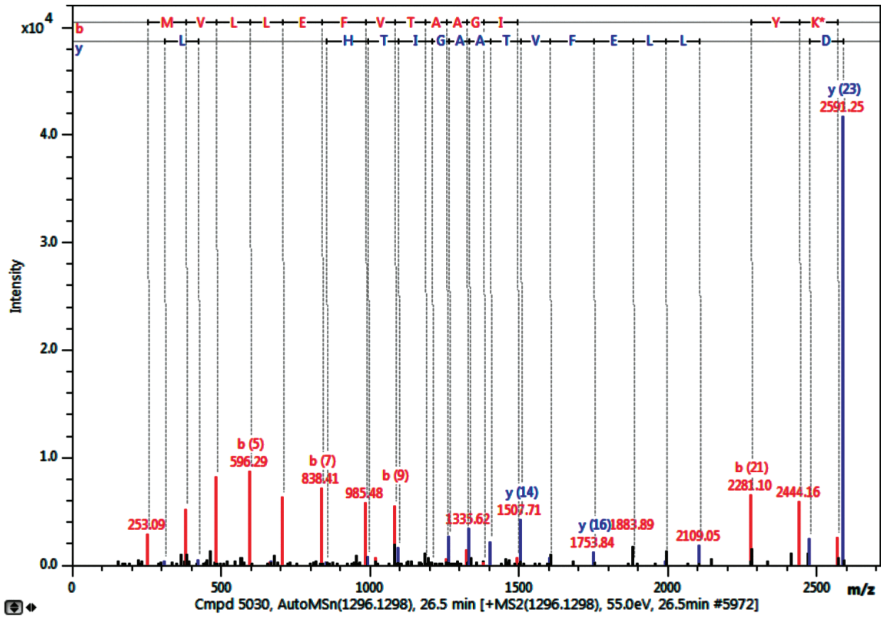

Supplementary Figure S4. Top-down sequencing of the H_{231}GMDELYK_{238} fragment of GFPuv form II using MALDI In-Source-Decay. Mass spectra for [M+3H]$^{3+}$ (top) and [M+2H]$^{2+}$ (bottom) ions are shown. Identified b-ions (red) and y-ions (blue), as well as unidentified signals (black), are indicated in the spectra.

```
GFPuv    MASKGEELFTGVVPILVELDGDVNGHKFSVSGEGEGDATYGKLTLKFICTTG-KLPVPWPTLVTTFSYGV
AvGFP    M-SKGEELFTGVVPILVELDGDVNGHKFSVSGEGEGDATYGKLTLKFICTTG-KLPVPWPTLVTTFSYGV
EGFP     MVSKGEELFTGVVPILVELDGDVNGHKFSVSGEGEGDATYGKLTLKFICTTG-KLPVPWPTLVTTLTYGV
AcGFP1   MVSKGAELFTGIVPILIELNGDVNGHKFSVSGEGEGDATYGKLTLKFICTTG-KLPVPWPTLVTTLSYGV
DsRed    M-RSSKNVIKEFMRFKVRMEGTVNGHEFEIEGEGEGRPYEGHNTVKLKVTKGGPLPFAWDILSPQFQYGS

GFPuv    QCFSRYPDHMKRHDFFKSAMPEGYVQERTISFKDDGNYKTRAEVKFEGDTLVNRIELKGIDFKEDGNILG
AvGFP    QCFSRYPDHMKQHDFFKSAMPEGYVQERTIFFKDDGNYKTRAEVKFEGDTLVNRIELKGIDFKEDGNILG
EGFP     QCFSRYPDHMKQHDFFKSAMPEGYVQERTIFFKDDGNYKTRAEVKFEGDTLVNRIELKGIDFKEDGNILG
AcGFP1   QCFSRYPDHMKQHDFFKSAMPEGYIQERTIFFEDDGNYKSRAEVKFEGDTLVNRIELTGTDFKEDGNILG
DsRed    KVYVKHPADIP--DYKKLSFPEGFKWERVMNFEDGGVVTVTQDSSLQDGCFIYKVKFIGVNFPSDGPVMQ

GFPuv    HK-LEYNYNSHNVYITADKQKNGIKANFKIRHNIEDGSVQLADHYQQNTPIGDGPVLLPDNHYLSTQSAL
AvGFP    HK-LEYNYNSHNVYIMADKQKNGIKVNFKIRHNIEDGSVQLADHYQQNTPIGDGPVLLPDNHYLSTQSAL
EGFP     HK-LEYNYNSHNVYIMADKQKNGIKVNFKIRHNIEDGSVQLADHYQQNTPIGDGPVLLPDNHYLSTQSAL
AcGFP1   NK-MEYNYNAHNVYIMTDKAKNGIKVNFKIRHNIEDGSVQLADHYQQNTPIGDGPVLLPDNHYLSTQSAL
DsRed    KKTMGWEASTERLYPR----DGVLKGEIHKALKLKDGGHYLVEF--KSIYMAKKPVQLPGYYYVDSKLDI

GFPuv    SKDPNEKRDHMVLLEFVTAAGITHGMDELYK
AvGFP    SKDPNEKRDHMVLLEFVTAAGITHGMDELYK
EGFP     SKDPNEKRDHMVLLEFVTAAGITLGMDELYK
AcGFP1   SKDPNEKRDHMIYFGFVTAAAITHGMDELYK
DsRed    TSHN-EDYTIVEQYERTEGRHHLFL------
```

Supplementary Figure S5. Structure-based amino acid sequence alignment of GFP and its related proteins. AvGFP and AcGFP are derived from *Aequorea victoria* and *Aequorea coerulescens*, respectively. UniProtKB accession numbers are as follows: AvGFP (P42212), EGFP (C5MKY7), and DsRed (Q9U6Y8). The GenBank accession number of GFPuv is AAC53663.1. The sequence of AcGFP was obtained from Clontech Laboratories Inc. (http://www.clontech.com). The sequences were aligned with the PROMALS3D multiple sequence and structure alignment server [1] based on the structures of GFPuv (PDB ID: 1B9C) and DsRed (PDB ID: 1GGX).

CBB

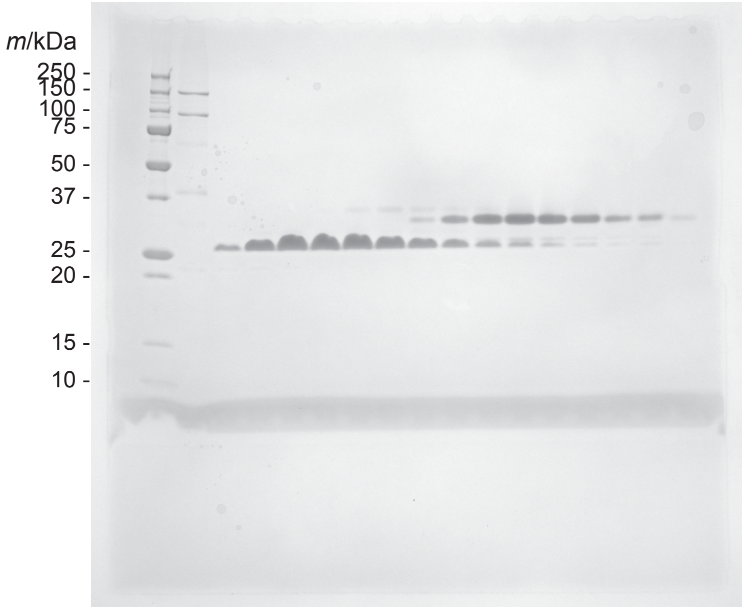

FL

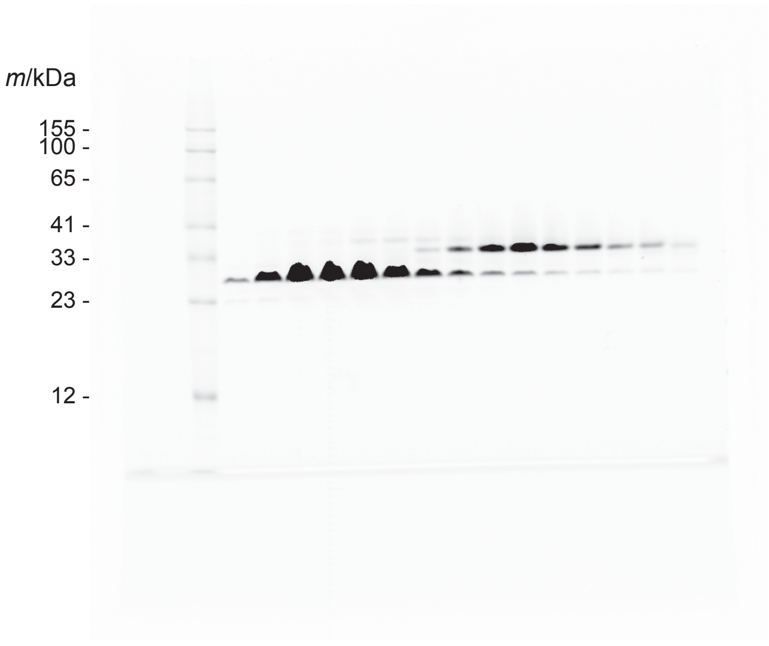

Supplementary Figure S6. The original gel images displayed on Figure 3A. Fluorescent bands (*bottom*) were visualized under ultraviolet light without any staining processes. After the detection of the fluorescent signal, the same gel was stained with CBB (*top*).

Reference for Supplementary Information

1 Pei, J., Kim, B. H. & Grishin, N. V. PROMALS3D: a tool for multiple protein sequence and structure alignments. *Nucleic acids research* **36**, 2295-2300, doi:10.1093/nar/gkn072 (2008).
